# Metagenomic Analysis of Lung Microbiome in Patients With Interstitial Lung Diseases and Sarcoidosis: An Experimental Study

**DOI:** 10.1002/hsr2.70328

**Published:** 2025-02-06

**Authors:** Suguru Takeuchi, Jun‐ichi Kawada, Atsushi Suzuki, Koji Sakamoto, Yuto Fukuda, Kazuhiro Horiba, Takako Suzuki, Yuka Torii, Yuichiro Shindo, Yoshinori Ito

**Affiliations:** ^1^ Department of Pediatrics Nagoya University Graduate School of Medicine Nagoya Japan; ^2^ Department of Pediatrics Fujita Health University School of Medicine Toyoake Japan; ^3^ Department of Respiratory Medicine Nagoya University Graduate School of Medicine Nagoya Japan; ^4^ Pathogen Genomics Center, National Institute of Infectious Diseases Tokyo Japan; ^5^ Department of Pediatrics Aichi Medical University Nagakute Japan

**Keywords:** acute exacerbation, interstitial lung diseases, lung microbiome, next‐generation sequencing, sarcoidosis

## Abstract

**Background and Aims:**

Interactions between the lung microbiome and pulmonary epithelium plays a pivotal role in shaping immunity in the lung. Idiopathic pulmonary fibrosis (IPF) is the most common interstitial lung disease (ILD). Some patients with IPF develop episodic acute exacerbations often associated with microbial dysbiosis in the lungs. This study aimed to investigate etiologic agents as well as the lung microbiome in patients with ILDs and sarcoidosis.

**Methods:**

This study analyzed 31 patients divided into the IPF (IPF‐stable, *n* = 12), acute exacerbation of ILDs (AE‐ILDs, *n* = 6), and sarcoidosis (*n* = 13) groups. Bronchoalveolar lavage fluid (BALF) samples were analyzed by RNA‐based metagenomic next‐generation sequencing (NGS) on an Illumina platform.

**Results:**

In total, 87 pathogens were detected using metagenomic NGS at the genus level. *Prevotella*, *Streptococcus*, and *Veillonella* dominated the BALF microbial communities, and sequence reads of these bacteria were abundant, especially in the sarcoidosis group. Conversely, only a small number of bacterial reads were detected in the AE‐ILDs group, and the overall proportion of microbial composition differed from that of the other groups. No significant difference was found in community diversity (α‐diversity) among the groups, whereas the structural similarity of the microflora (β‐diversity) differed significantly between the AE‐ILDs and sarcoidosis groups.

**Conclusions:**

Bacterial sequence reads in BALF were smaller in both the IPF‐stable and AE‐ILD groups than in the sarcoidosis group. Dysbiosis in the lung microbiome has been observed in patients with AE‐ILD and may be related to the progression of inflammation.

## 1 Introduction

Idiopathic pulmonary fibrosis (IPF) is the most common type of interstitial lung disease (ILD) and is characterized by chronic and progressive fibrotic ILD of unknown etiology [1, 2]. The clinical course of IPF varies, and most patients experience a gradual worsening of pulmonary function over time. In contrast, some patients with IPF develop episodic acute respiratory deterioration, termed acute exacerbation of IPF (AE‐IPF) [2, 3, 4, 5, 6]. AE‐IPF is pathophysiologically characterized by rapid respiratory failure with the appearance of a new invasion shadow in the lungs during IPF's chronic course [6, 7]. Although viral infection is considered a possible trigger of AE‐IPF, the role of viruses in AE‐IPF remains inconclusive [6, 7, 8].

With advances in molecular sequencing technology, culture‐independent microbiology has revealed that the lungs, previously considered sterile, harbor a complex and dynamic community of microbes. The interactions between the lung microbiome and pulmonary epithelium play a pivotal role in shaping immunity in the lung [9]. Furthermore, each respiratory disease may have unique lung microbial signatures, and these signatures could be used as diagnostic markers [10]. Microbiome analysis of bronchoalveolar lavage fluid (BALF) samples revealed the microbiome's composition and potential role in the ILDs' pathogenesis [11, 12, 13, 14, 15]. Several studies have revealed an association between the lung microbiome and disease progression in ILDs; however, only a few have investigated BALF samples obtained during acute exacerbation cases [16, 17, 18]. Although it is currently unclear whether increased bacterial burden reflects active infection, microbial dysbiosis is related to disease outcomes in patients with AE‐IPF.

Sarcoidosis is an unexplained systemic granulomatous disease that may cause secondary interstitial pneumonia [19]. Similar to other ILDs, sarcoidosis appears to be associated with an aberrant immune response to antigenic or inflammatory triggers [19, 20, 21]. Although there is no established microbial cause of sarcoidosis, some bacterial candidates have been examined as potential triggers [20]. Recently, Clarke et al. performed an intensive metagenomic investigation of multiple sarcoidosis samples, including bronchoalveolar BALF, and identified several potential candidates [21].

BALF is considered a suitable sample for analysis of the lung microbiome because of its sterile nature and low risk of contamination by resident bacteria in the oral cavity [22]. In previous studies on the lung microbiome, 16S rRNA gene sequencing analysis has been commonly used [11, 12, 13, 14, 16, 17]. However, targeted 16S rRNA sequencing is exclusively used for bacterial profiling and only provides microbiome data at the family and genus levels. Compared to the 16S rRNA method, metagenomic next‐generation sequencing (NGS) allows us to obtain complete genomic information for all microorganisms, including bacteria, viruses, and fungi [23, 24]. Furthermore, RNA‐based metagenomic NGS provides a transcriptionally active microbiome profile [25]. We previously demonstrated the efficacy of RNA‐based metagenomic NGS for identifying bacterial and viral pathogens in BALF samples from pediatric patients with respiratory failure [23, 26]. Our RNA‐based metagenomic NGS method can detect low‐abundance pathogens in BALF with high sensitivity using small sample volume. This study aimed to analyze and investigate the etiologic agents and lung microbiomes in patients with ILDs and sarcoidosis using metagenomic NGS.

## 2 Methods

### 2.1 Patients and Samples

BALF samples were obtained from patients categorized into three groups: stable IPF (IPF‐stable), acute exacerbation of ILDs (AE‐ILD), and sarcoidosis. The definition and diagnosis of IPF, AE‐ILDs, and sarcoidosis were based on established criteria as previously described [2, 11, 19]. All BALF samples were obtained from the patients during a routine clinical examination. In the AE‐ILDs group, BALF was obtained within 3 days of admission. In the sarcoidosis group, BALF was obtained from patients who underwent initial diagnostic bronchoscopy. All the samples were cryopreserved at −80°C until use. Furthermore, no‐template control (NTC) samples (*n* = 3) prepared from distilled water were analyzed using the procedure described below.

### 2.2 Ethics Approval and Consent to Participate

This study was performed in accordance with the principles of the Declaration of Helsinki and approved by the Institutional Review Board of Nagoya University Graduate School of Medicine (approval number: 2015‐0236‐9). Written informed consent was obtained from all the patients.

### 2.3 Library Preparation and Sequencing

RNA was extracted from 200‐µL BALF samples using the NucleoSpin RNA blood kit (MACHEREY‐NAGEL, Düren, Germany). Complementary DNA (cDNA) was synthesized from extracted RNA using the REPLI‐g WTA Single‐Cell Kit (Qiagen, Hilden, Germany). The Nextera XT DNA sample preparation kit (Illumina, San Diego, CA, USA) was used to prepare libraries from the generated cDNA. Library quality was analyzed using an Agilent 2100 Bioanalyzer (Agilent Technologies, Santa Clara, CA, USA), a Qubit double‐stranded DNA high‐sensitivity assay kit (Thermo Fisher Scientific, Waltham, MA, USA), and a QX200 droplet digital polymerase chain reaction (PCR) system (Bio‐Rad, Richmond, CA, USA). The fragment size range and concentration of each library were 363–1057 bp (median, 650 bp) and 11.7–141.6 nM (median, 39.8 nM), respectively. Indexed libraries were pooled and sequenced using a HiSeq. 2500 (Illumina).

### 2.4 Next‐Generation Sequencing Data Processing and Analysis

The FASTQ files were uploaded to the cloud‐computing metagenomic pipeline MePIC version 2.0 (National Institute of Infectious Disease, Tokyo, Japan) [27] to analyze the sequence data. Briefly, unnecessary adapter sequences, low‐quality bases (Q‐score cut‐off: 20), and short reads (length cut‐off: 50) were trimmed. Human‐derived reads were then detected using the BWA program and removed from the downstream analysis. For the remaining reads, MEGABLAST was used to search for sequences similar to those registered in the National Center for Biotechnology Information nucleotide database (E‐value cut‐off, 1e‐30). Finally, the search results were summarized in terms of taxonomic information using MEGAN6 (University of Tübingen, Tübingen, Germany) [28]. To exclude common nonpathogenic contaminants that may have originated from the environment, laboratory reagents, or due to cross‐contamination, the pathogen‐derived reads were considered significant when the threshold of the reads per million ratio metrics (RPM‐r) of ≥ 10 was satisfied as previously described [23, 29, 30, 31]. The RPM‐r was calculated as the RPM corresponding to a given species in the clinical sample divided by the RPM in the NTC.

### 2.5 Statistics

Statistical analyses were performed using the Statistical Package for the Social Sciences version 24.0 (IBM, Chicago, IL, USA). Statistical differences between two and three groups were evaluated using the Mann–Whitney U and Kruskal–Wallis tests, respectively. *p* < 0.05 were considered statistically significant. The differences in distribution of microbial composition at the genus level within a sample (*α*‐diversity) were assessed by Shannon diversity using the PAST version 3.24 software [32]. The structural similarity of the microflora (*β*‐diversity) between the groups in this study was visualized using principal coordinate analysis (PCoA) based on the Bray–Curtis dissimilarity distance and tested using permutational multivariate analysis of variance in PAST version 3.24 software.

## 3 Results

### 3.1 Patient Characteristics

This study analyzed 31 patients divided into the IPF‐stable (*n* = 12), AE‐ILDs (*n* = 6), and sarcoidosis (*n* = 13) groups. The patients' clinical characteristics are summarized in Table 1.

**Table 1:** Patient characteristics.

	IPF‐stable (*n* = 12)	AE‐ILDs (*n* = 6)	Sarcoidosis (*n* = 13)	*p* value*
Age, years	66.8 ( ± 7.1)	70.2 ( ± 8.6)	57.8 ( ± 16.7)	0.373
Sex (Male/Female)	10/2	5/1	4/9	—
Pack‐years	42.5 ( ± 34.3)	28.9 ( ± 17.8)	10.3 ( ± 16.1)	**0.012** †
FEV_1_, %	80.7 ( ± 19.5)	70.4 ( ± 14.6)	95.6 ( ± 25.1)	0.037
FVC, %	79.9 ( ± 17.8)	68.0 ( ± 13.1)	103.6 ( ± 22.2)	**0.006** ‡
DL_CO_, %	69.4 ( ± 20.1)	55.3 ( ± 19.4)	86.7 ( ± 26.7)	0.097
WBC/μL	7058 ( ± 1951)	9866 ( ± 9682)	6346 ( ± 2094)	0.685
CRP, mg/dL	0.27 ( ± 0.18)	7.05 ( ± 6.60)	0.20 ( ± 0.23)	**0.008** §
KL‐6, U/mL	970 ( ± 458)	1514 ( ± 741)	1665 ( ± 3243)	0.044
BALF TCC, ×10^5^/mL	2.736 ( ± 1.558)	3.633 ( ± 1.717)	1.925 ( ± 1.432)	0.055
BALF neutrophils, %	2.2 ( ± 2.2)	24.8 ( ± 34.0)	2.5 ( ± 4.9)	**0.023** ||
BALF lymphocyte, %	14.5 ( ± 16.0)	21.3 ( ± 33.0)	24.1 ( ± 16.2)	0.143
CD4/8	1.53 ( ± 0.64)	2.58 ( ± 1.06)	5.42 ( ± 3.29)	**0.001** ¶

*Note:* Data are represented as mean ( ± standard deviation), unless otherwise noted. Bold values indicate statistically significance *p* < 0.05.

Abbreviations: AE‐ILDs, acute exacerbation of interstitial lung diseases; BALF, bronchoalveolar lavage fluid; CRP, C‐reactive protein; DL_CO_, Diffusing capacity of lung carbon monoxide; FEV1, forced expiratory volume in the first second; FVC, forced vital capacity; KL‐6, Krebs von den Lungen‐6; IPF, idiopathic pulmonary fibrosis; TCC, total cell counts; WBC, white blood cell.

* *p* value is comparison between the IPF‐stable, AE‐ILDs, and sarcoidosis groups.

^†^ IPF‐stable versus AE‐ILDs: *p* = 0.35; IPF‐stable versus sarcoidosis: *p* = 0.005; AE‐ILDs versus sarcoidosis: *p* = 0.06.

^‡^ IPF‐stable versus AE‐ILDs: *p* = 0.21; IPF‐stable versus sarcoidosis: *p* = 0.008; AE‐ILDs versus sarcoidosis: *p* = 0.02.

^§^ IPF‐stable versus AE‐ILDs: *p* = 0.02; IPF‐stable versus sarcoidosis: *p* = 0.23; AE‐ILDs versus sarcoidosis: *p* = 0.004.

^||^ IPF‐stable versus AE‐ILDs: *p* = 0.04; IPF‐stable versus sarcoidosis: *p* = 0.14; AE‐ILDs versus sarcoidosis: *p* = 0.005.

^¶^ IPF‐stable versus AE‐ILDs: *p* = 0.057; IPF‐stable versus sarcoidosis: *p* = 0.001; AE‐ILDs versus sarcoidosis: *p* = 0.054.

### 3.2 Sequence Results

An average of 10,778,342 reads per sample were obtained from each BALF sample. After removing low‐quality reads and adapter sequences, 8,379,983 reads (77.7%) remained and were analyzed. Of these, 60.2% were derived from the human genome, and 0.8% were annotated to bacterial and/or viral sequences with high confidence (bit score ≥ 250). Conversely, a majority of the remaining 39% of reads consisted of bacterial and/or viral sequences with low confidence (bit score < 250), along with sequences classified as nonhuman vertebrate based on BLAST analysis, and sequences for which we obtained no BLAST hits. Details of the sequencing data are listed in Table S1. In total, 90 and 87 pathogens met the positive detection threshold at the species and genus levels, respectively (Tables S2 and S3). Among all genera, *Pseudomonas* and *Streptococcus* were the most diverse, comprising 15 and 13 species, respectively. However, the relationship between specific pathogens at the species level and the patient groups remains unclear. Therefore, further analyses were performed at the genus level to investigate the relationship between the lung microbiome and patient groups. No significant viral sequences were detected in any samples.

### 3.3 Lung Microbiome in Interstitial Lung Diseases and Sarcoidosis Patients at the Genus Level

**Figure 1:**
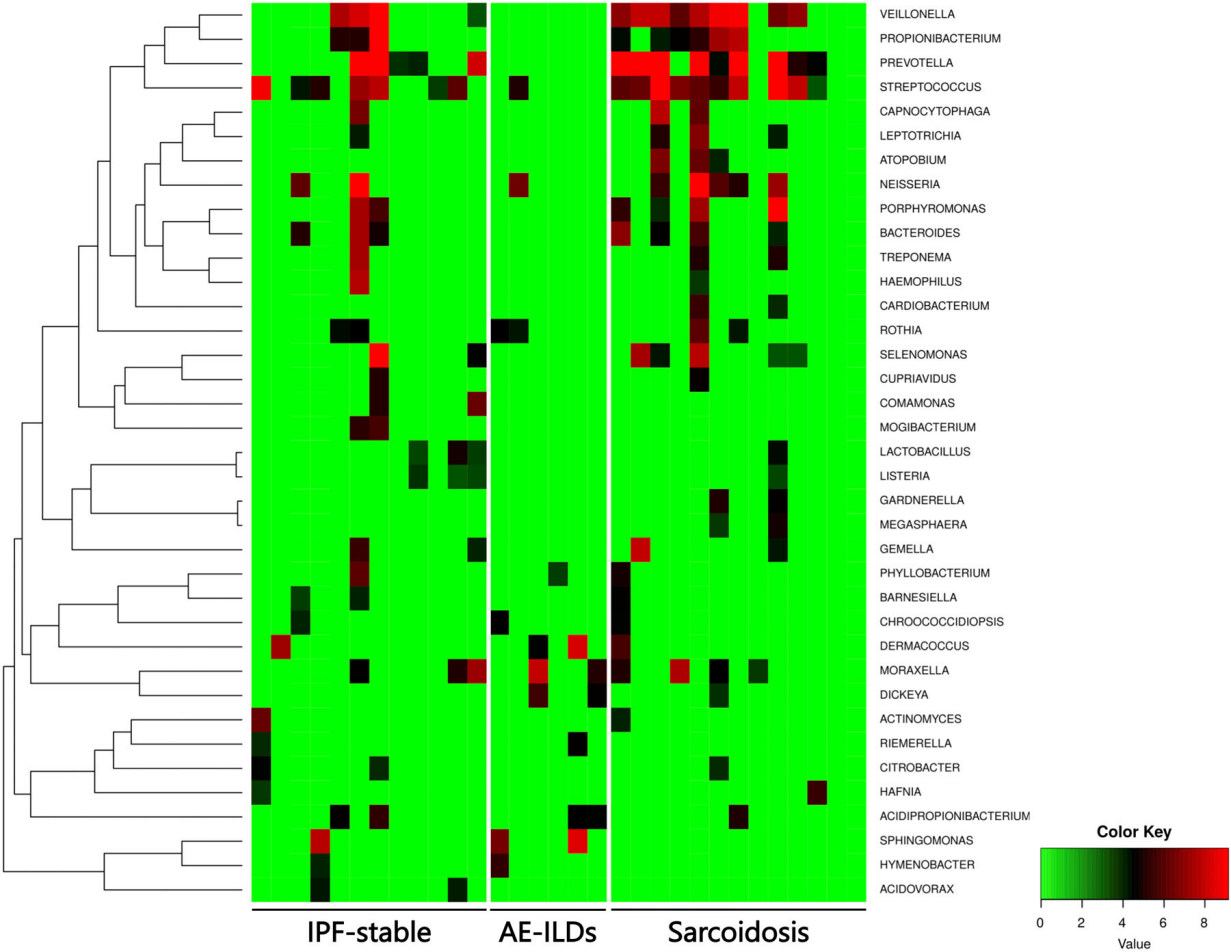
Frequency of the normalized abundance of bacterial reads grouped by disease status. The frequency of the normalized abundance of bacterial reads at the genus level was summarized in a heat map to compare the base microbiome of each sample. AE‐ILDs, acute exacerbation of interstitial lung disease; IPF‐stable, stable state of idiopathic pulmonary fibrosis.

**Figure 2:**
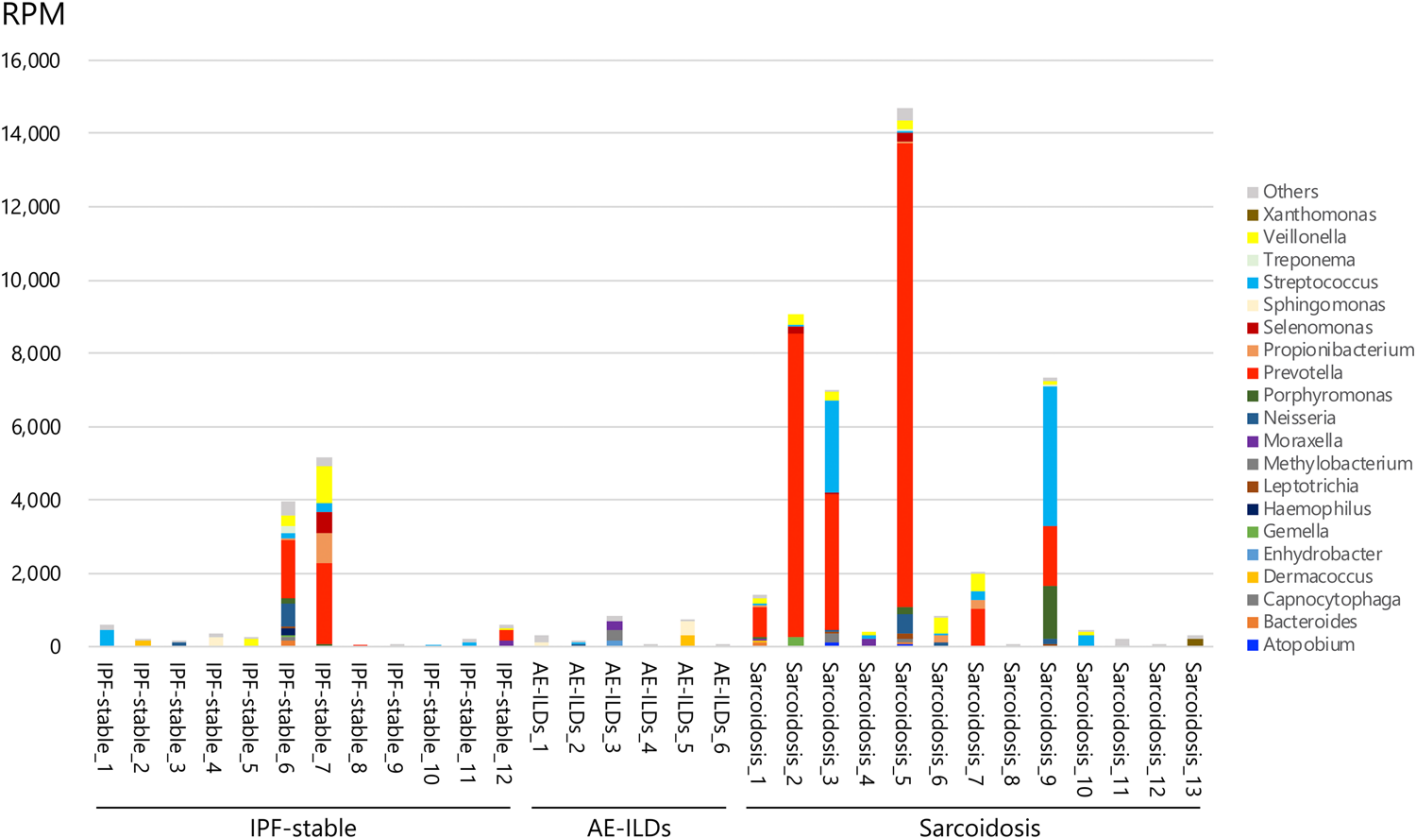
Comparison of the microbial composition at the genus level. Microbial composition based on the 20 most prominent genera is shown as stacked bar graphs. AE‐ILDs, acute exacerbation of interstitial lung disease; IPF‐stable, stable state of idiopathic pulmonary fibrosis; RPM, reads per million.

**Figure 3:**
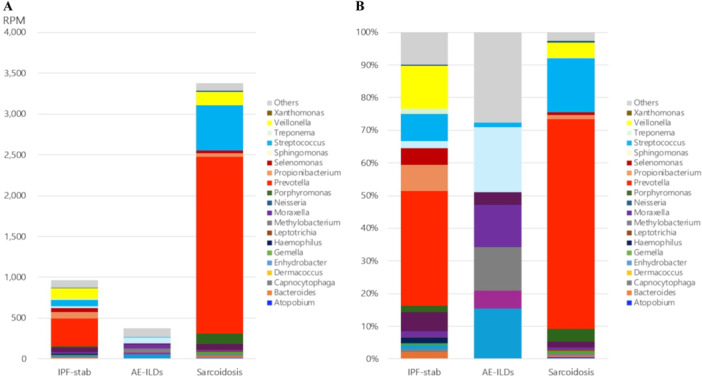
Comparison of the microbial distribution at the genus level. The distribution of microbial composition at the genus level in each patient group is shown as the average of bacterial reads (A) and relative abundance (B). AE‐ILDs: acute exacerbation of interstitial lung disease; IPF‐stable, stable state of idiopathic pulmonary fibrosis; RPM, reads per million.

The frequency of the normalized abundance of bacterial reads at the genus level is summarized in a heat map to compare the base microbiome of each sample (Figure 1). The genus composition of each patient is shown in Figure 2 and Figure S1. Twenty prominent genera accounted for 94.9% of the total bacterial reads in all the patients. Among these, the *Prevotella*, *Streptococcus*, and *Veillonella* genera dominated the BALF microbial communities, especially in the IPF‐stable and sarcoidosis groups. In contrast, only a small number of bacterial sequence reads were detected in the AE‐ILDs group, and the overall microbial composition differed from that of the other groups (Figure 3). Compared to the AE‐ILDs group, bacterial sequence reads were larger in the IPF‐stable and sarcoidosis groups, but there was no significant difference among the three groups. The phylum composition of each patient (Figures S2 and S3) and group (Figure S4) is shown. No significant differences in number of bacterial sequence reads at the phylum level were observed among three groups (Figure S4).

**Figure 4:**
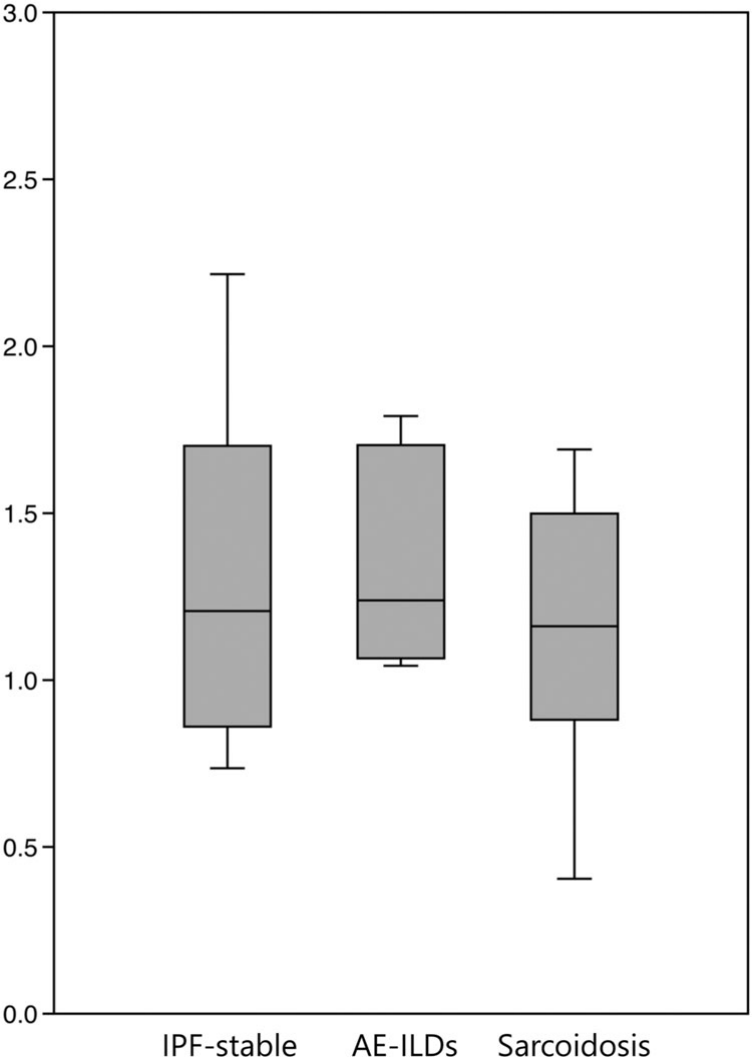
Comparison of the microbial community diversity. The α‐diversity at the genus level is represented by a box plot. Box plots are expressed as median ± interquartile ranges. There was no significant difference in the α‐diversity among the patient groups as determined by the Kruskal Wallis test (*p* = 0.596). AE‐ILDs, acute exacerbation of interstitial lung disease; IPF‐stable, stable state of idiopathic pulmonary fibrosis.

**Figure 5:**
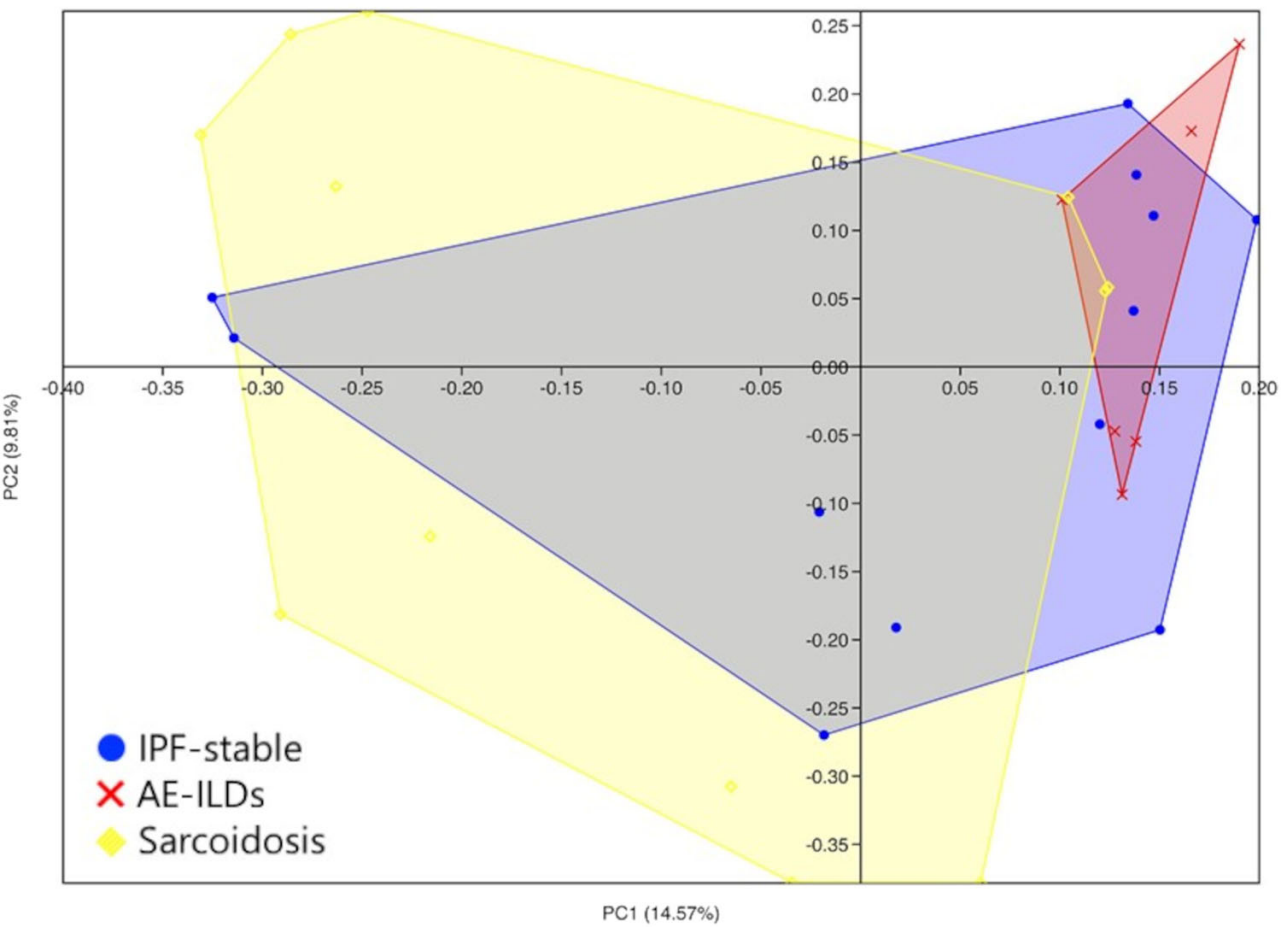
Two‐dimensional principal coordinate analysis plot of the microbial community. The comparison of the structural similarity of the microflora (*β*‐diversity) at the genus level between the groups was visualized using the principal coordinate analysis (PCoA) based on the Bray‐Curtis dissimilarity distance. Black circle, cross mark, and diamond represent IPF‐stable, AE‐ILDs, and sarcoidosis samples, respectively. AE‐ILDs, acute exacerbation of interstitial lung disease; IPF‐stable, stable state of idiopathic pulmonary fibrosis.

**Figure 6:**
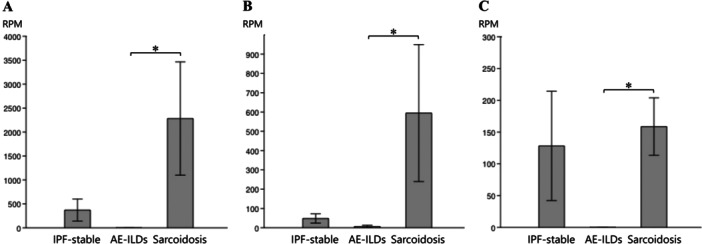
Differences in the component bacteria at the genus level among the patient groups. The average number of sequence reads annotated to each pathogen was compared among the patient groups. The average number of reads annotated to *Prevotella* (A), *Streptococcus* (B), and *Veillonella* (C) is shown. Data are represented as mean ± standard deviation. *p < 0.05 by Mann‐Whitney U‐test. AE‐ILDs, acute exacerbation of interstitial lung disease; IPF, idiopathic pulmonary fibrosis; IPF‐stable, stable state of IPF; RPM, reads per million.

When comparing the bacterial community diversity among the groups, no significant difference was found in the α‐diversity at the genus level (Figure 4). Furthermore, no significant correlation was observed between pulmonary function test results and α‐diversity (data not shown). In contrast, the structural similarity of the microflora (β‐diversity) at the genus level differed significantly between the AE‐ILDs and sarcoidosis groups in the PCoA analysis (*p* = 0.010, Figure 5). The bacterial sequence reads of *Prevotella*, *Streptococcus*, and *Veillonella* were significantly higher in the sarcoidosis group than in the AE‐ILDs group (Figure 6). This may have contributed to the significant difference in the β‐diversity. In contrast, there were no distinct differences in microbial composition between the AE‐ILDs and IPF‐stable groups and the IPF‐stable and sarcoidosis groups. On the other hand, no significant difference was found in the α‐ and β‐diversity at the species level among the patient groups because identification at the species levels in this study was not sufficiently adequate (data not shown).

## 4 Discussion

Recent studies have shown that human lungs are colonized by various commensal bacterial species that may play a role in both health and disease [9]. We performed a comprehensive pathogen analysis of BALF samples from patients with ILDs and sarcoidosis using RNA‐based metagenomic NGS. Abundant reads of bacterial species, such as *Prevotella* and *Streptococcus*, were detected at the species level in some patients in the IPF‐stable and sarcoidosis groups. However, only a few bacterial reads were detected in the AE‐ILDs group. Although positive cutoff values for detecting causative pathogens by metagenomic NGS have not been established, bacterial reads detected in patients with AE‐ILDs were not considered significant based on our previous studies [23, 26].

The possible role of viral infection in AE‐IPF has been reported in previous studies [6, 7, 8, 33, 34, 35, 36]. Wootton et al. demonstrated that 9% of AE‐IPF BALF samples tested positive for common respiratory viruses, whereas all IPF‐stable samples tested negative [8]. They also revealed that the torque teno virus was significantly more common in patients with AE‐IPF than in those with IPF‐stable. However, the pathogenic significance of viral infections in the progression of AE‐IPF remains inconclusive [6, 7, 8]. Most previous studies have used PCR to determine the etiologic agents of AE‐IPF. Although PCR is currently considered the most sensitive method, it can identify only a defined set of candidate pathogens. In contrast, metagenomic NGS can detect pathogen‐derived reads without specific primers, and our previous studies have demonstrated the comprehensive and efficient detection of RNA viruses in BALF samples [23, 26]. However, no significant viruses were detected in any of the samples in the present study, including the AE‐ILDs samples.

Although there were no significant differences in the average number of bacterial reads or α‐diversity at the genus level among the groups in this study, abundant bacterial reads were detected in some patients in the IPF stable and sarcoidosis groups. Furthermore, there was a distinct difference in the structural similarity of the lung microbiomes between the AE‐ILDs and sarcoidosis groups. In particular, *Prevotella*, *Veillonella*, and *Streptococcus* were more abundant in the sarcoidosis group than in the AE‐ILDs group. These results were in line with the recent blood microbiome analysis by Hodzhev showing that *Prevotella* and *Veillonella* exhibited high abundance in the sarcoidosis [37]. Role of these bacteria in sarcoidosis has not been fully understood. *Prevotella* may play a role in the development of sarcoidosis by contributing to the formation of granuloma through immune system activation. Moreover, *Veillonella* species produce lactic and other fatty acids, triggering inflammatory response leading to granuloma formation [37]. The composition of these bacteria corroborates that of the lung microbiome of patients with IPF and sarcoidosis, as shown in previous studies [11, 20]. In contrast, these bacteria have also been reported to constitute normal lung flora in healthy adults [38]. However, significant reads of *Cutibacterium acnes*, which have been implicated as possible etiological agents of sarcoidosis, were not detected in any patient [39]. Our results suggest that inflammation in AE‐ILDs may have affected the constitution of the lung microbiome or that dysbiosis may be related to the progression of IPF, as shown in previous studies [16, 17, 18]. Furthermore, an association between alterations in the lung microbiome and inflammation has been demonstrated in mouse models [40, 41]. Dysbiosis may induce an aberrant immune response, contributing to inflammation and fibrosis seen in PF‐ILD. Thus, lung dysbiosis may be a potential therapeutic target for IPF [42].

This study has several limitations. First, it was retrospective and nonrandomized, indicating the potential for sampling biases. Second, the sample size was relatively small because of the invasiveness of the procedure, especially in patients with AE‐ILDs, which restricted the statistical power. Third, the lung microbiomes of healthy participants were not analyzed owing to ethical issues. Finally, antibiotic therapy administered before sampling may have affected these results. Five of the six AE‐ILDs samples were collected after the commencement of antibiotics because bacterial infection could not be initially ruled out in these cases. Moreover, comparing the changes in the lung microbiota before and after ILD treatment is essential.

## 5 Conclusions

This is the first study to investigate the etiological agents and lung microbiome in patients with ILDs using RNA‐based metagenomic NGS, which can provide a transcriptionally active microbiome profile. No potentially exacerbating viral or bacterial pathogens were detected in the patients with AE‐ILDs. Lung microbiome dysbiosis was observed in AE‐ILDs and may be related to the progression of inflammation. Future prospective investigations with larger sample sizes are needed to clarify the contribution of infectious etiologies and lung microbiomes to ILDs.

## Author Contributions

Conceptualization: Jun‐ichi Kawada, Koji Sakamoto, Yuichiro Shindo, and Yoshinori Ito. Data curation: Suguru Takeuchi and Atsushi Suzuki. Formal analysis: Suguru Takeuchi, Jun‐ichi Kawada, Yuto Fukuda, and Kazuhiro Horiba. Funding acquisition: Jun‐ichi Kawada and Yoshinori Ito. Investigation: Suguru Takeuchi, Atsushi Suzuki, Kazuhiro Horiba, Takako Suzuki, and Yuka Torii. Supervision: Koji Sakamoto, Yuichiro Shindo, and Yoshinori Ito. Validation: Jun‐ichi Kawada and Yoshinori Ito. Visualization: Suguru Takeuchi, Yuto Fukuda, and Jun‐ichi Kawada. Writing–original draft: Suguru Takeuchi and Jun‐ichi Kawada. Writing–review and editing: Yoshinori Ito.

## Conflicts of Interest

The authors declare no conflicts of interest.

### 1 Transparency statement

J.K. affirms that this manuscript is an honest, accurate, and transparent account of the study being reported; that no important aspects of the study have been omitted; and that any discrepancies from the study as planned have been explained.

## Supporting information

Supporting information.

Supporting information.

Supporting information.

Supporting information.

Supporting information.

Supporting information.

## Data Availability

The data that support the findings of this study are available from the corresponding author, J.K., upon reasonable request. The data are not publicly available due to their containing information that could compromise the privacy of research participants. All authors have read and approved the final version of the manuscript. Jun‐ichi Kawada has full access to all of the data in this study and takes complete responsibility for the integrity of the data and the accuracy of the data analysis.
