# Supplementary figures and images for: Metagenomic Analysis of Lung Microbiome in Patients With Interstitial Lung Diseases and Sarcoidosis: An Experimental Study
Source: Health Sci Rep. 2025 Feb 6;8(2):e70328. doi: 10.1002/hsr2.70328 (PMC11803077; doi:10.1002/hsr2.70328)

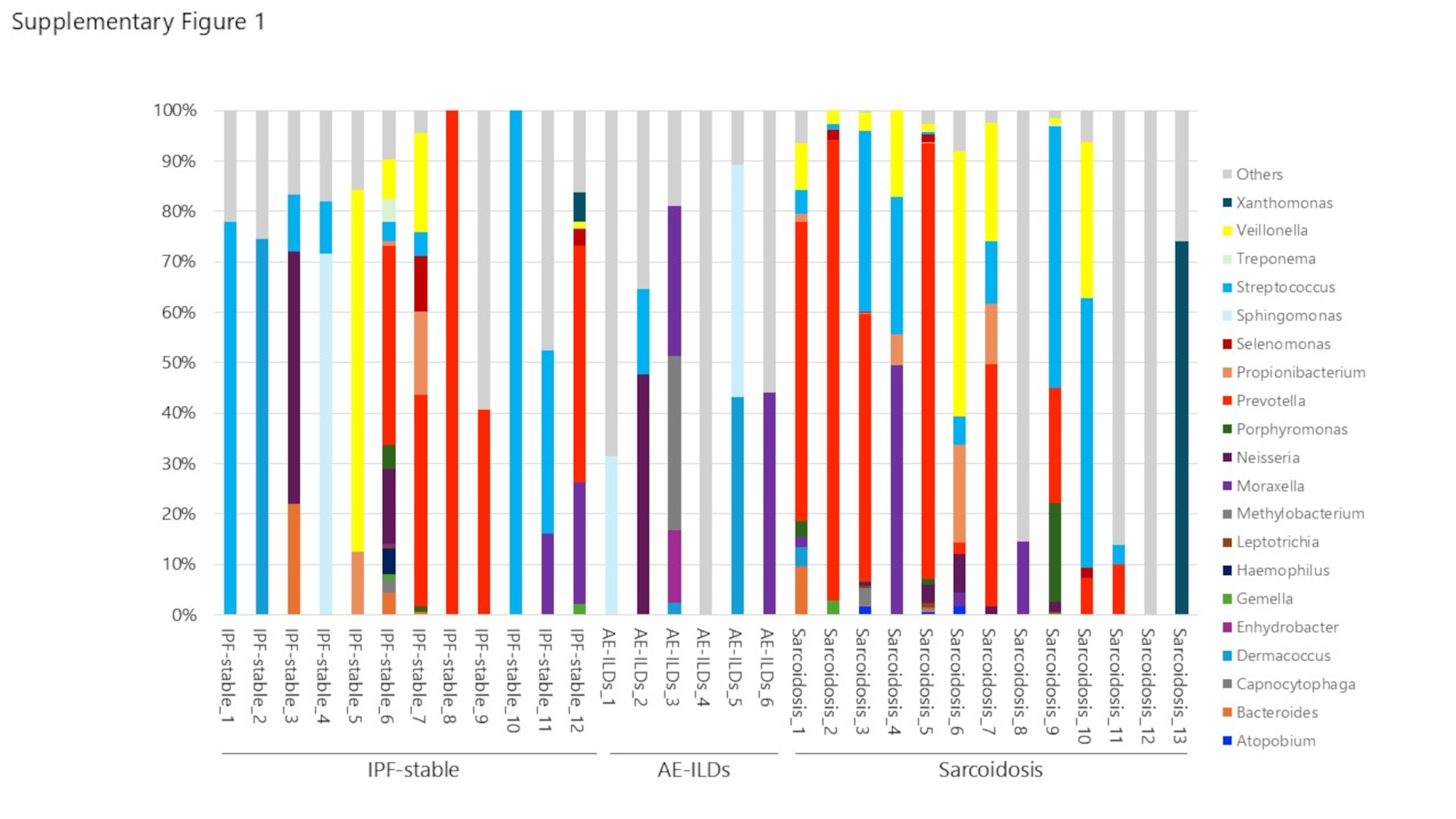

Supplement: Supplementary file 1 — Supporting information. [file HSR2-8-e70328-s004.JPG]

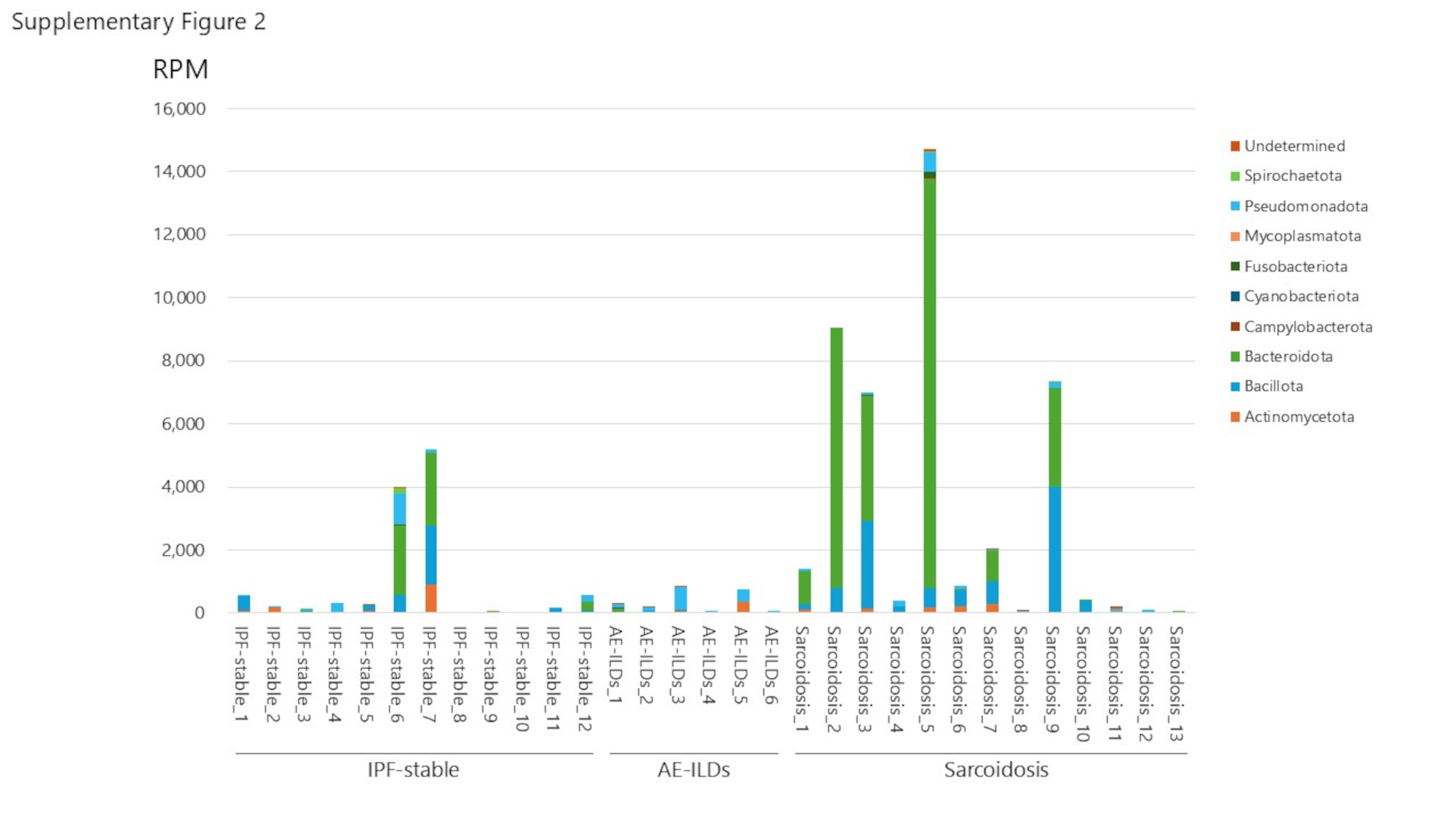

Supplement: Supplementary file 2 — Supporting information. [file HSR2-8-e70328-s006.JPG]

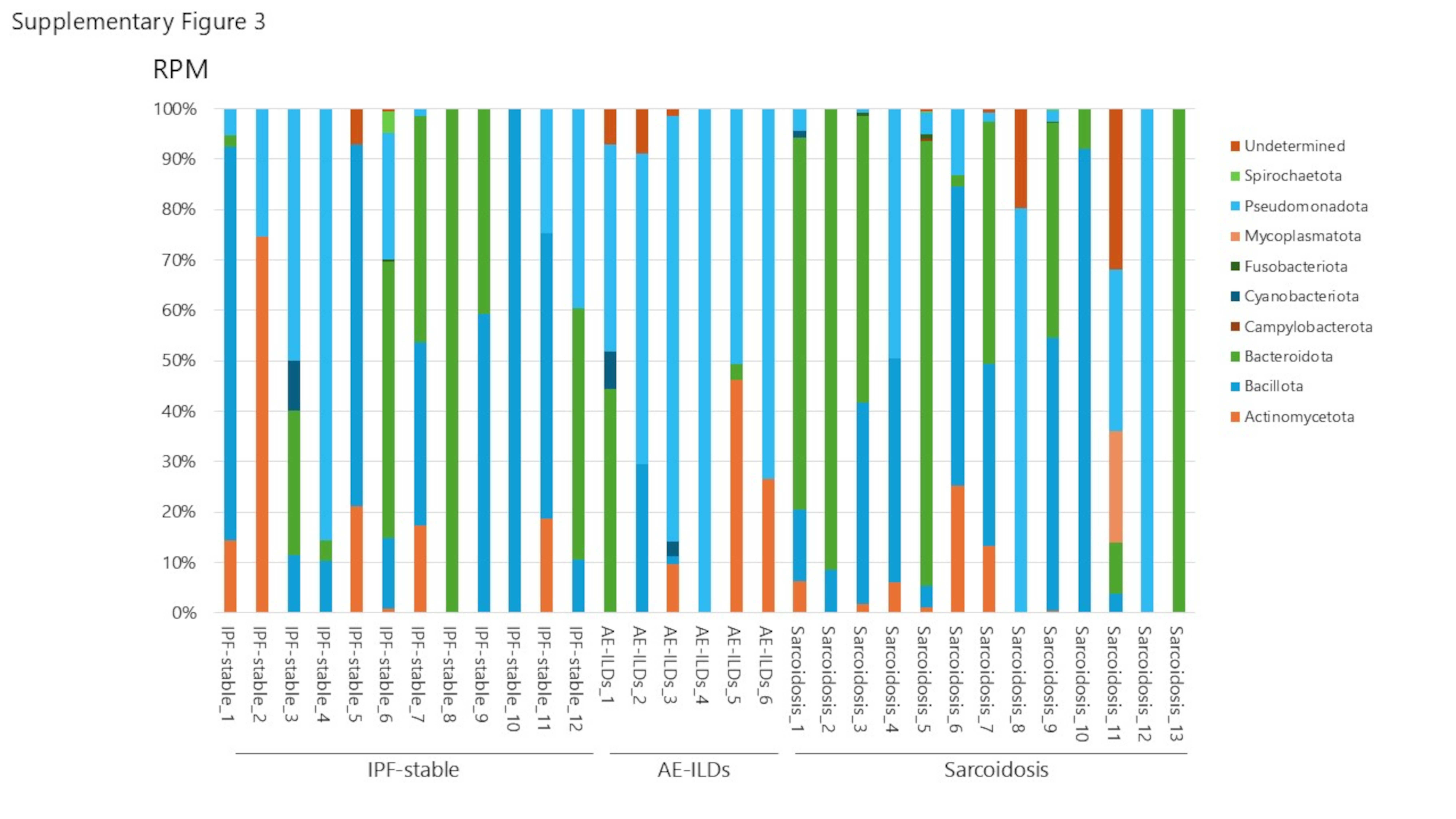

Supplement: Supplementary file 3 — Supporting information. [file HSR2-8-e70328-s002.JPG]

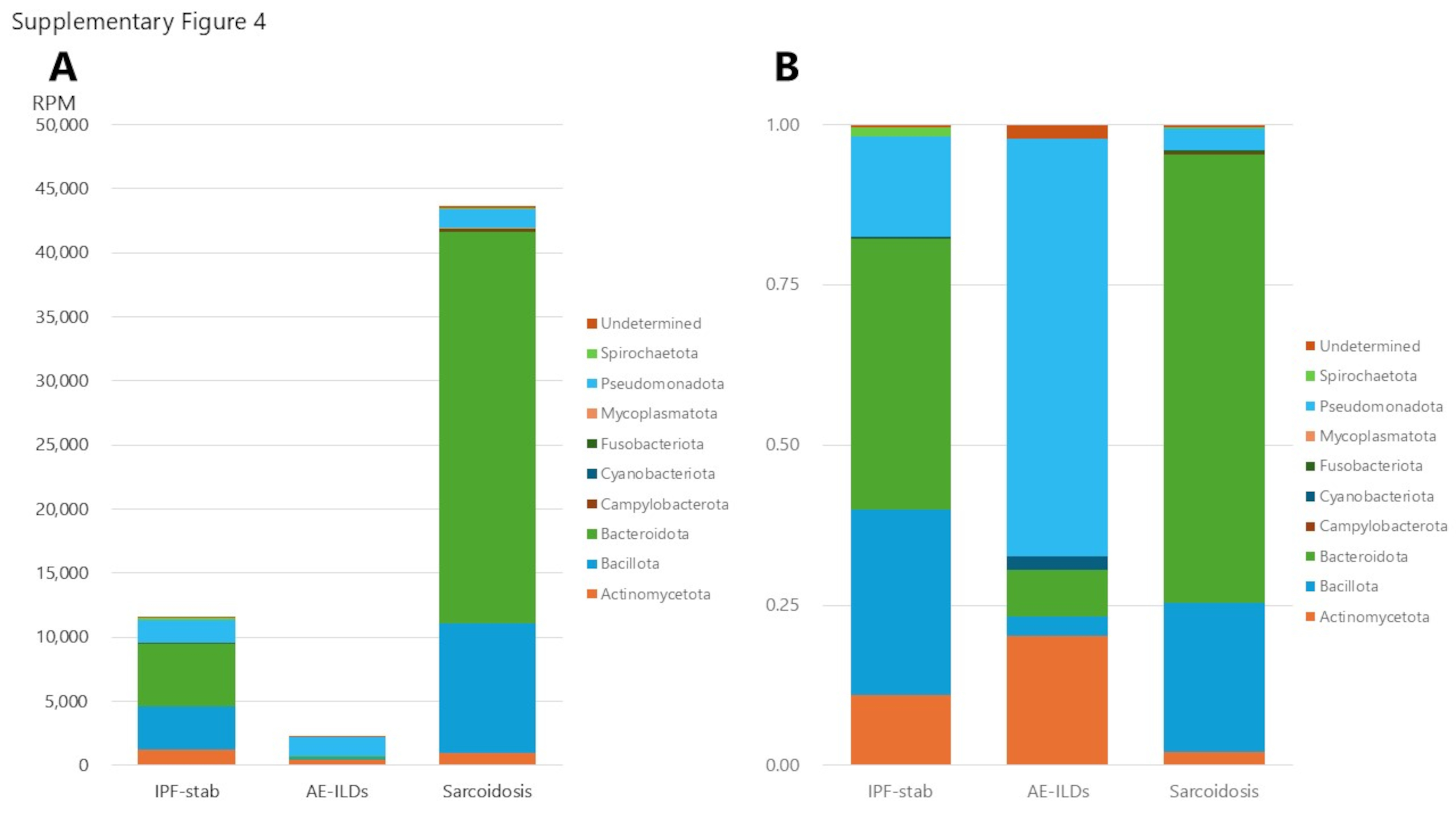

Supplement: Supplementary file 4 — Supporting information. [file HSR2-8-e70328-s003.JPG]
