# Supplementary material for: Metagenomic Analysis of Lung Microbiome in Patients With Interstitial Lung Diseases and Sarcoidosis: An Experimental Study
Source: Health Sci Rep. 2025 Feb 6;8(2):e70328. doi: 10.1002/hsr2.70328 (PMC11803077; doi:10.1002/hsr2.70328)
Supplement: Supplementary file 6 — Supporting information. [file HSR2-8-e70328-s005.docx]

**Figure S1. Comparison of the microbial composition at the genus level**

Microbial composition based on the 20 most prominent genera is shown as relative abundance.

IPF-stable: stable state of idiopathic pulmonary fibrosis; AE-ILDs: acute exacerbation of interstitial lung disease; RPM: reads per million

**Figure S2. Comparison of the microbial composition at the phylum level**

Microbial composition based on the phyla is shown as stacked bar graphs.

IPF-stable: stable state of idiopathic pulmonary fibrosis; AE-ILDs: acute exacerbation of interstitial lung disease; RPM: reads per million

**Figure S3. Comparison of the microbial composition at the phylum level**

Microbial composition based on the phyla is shown as relative abundance.

IPF-stable: stable state of idiopathic pulmonary fibrosis; AE-ILDs: acute exacerbation of interstitial lung disease; RPM: reads per million

**Figure S4. Comparison of the microbial distribution at the phylum level**

The distribution of microbial composition at the phylum level in each patient group is shown as the average of bacterial reads (A) and relative abundance (B).

IPF-stable: stable state of idiopathic pulmonary fibrosis; AE-ILDs: acute exacerbation of interstitial lung disease; RPM: reads per million
